# Bone Regeneration of a 3D-Printed Alloplastic and Particulate Xenogenic Graft with rhBMP-2

**DOI:** 10.3390/ijms222212518

**Published:** 2021-11-20

**Authors:** Ji-In Ryu, Byoung-Eun Yang, Sang-Min Yi, Hyo-Geun Choi, Sung-Woon On, Seok-Jin Hong, Ho-Kyung Lim, Soo-Hwan Byun

**Affiliations:** 1 Department of Pediatric Dentistry, Sacred Heart Hospital, Hallym University College of Medicine, Anyang 14068, Korea; rji0112@naver.com; 2 Graduate School of Clinical Dentistry, Hallym University, Chuncheon 24252, Korea; face@hallym.or.kr (B.-E.Y.); queen21c@gmail.com (S.-M.Y.); drummer0908@hanmail.net (S.-W.O.); 3 Research Center of Clinical Dentistry, Clinical Dentistry Graduate School, Hallym University, Chuncheon 24252, Korea; pupen@naver.com (H.-G.C.); enthsj@hanmail.net (S.-J.H.); 4 Department of Oral and Maxillofacial Surgery, Sacred Heart Hospital, Hallym University College of Medicine, Anyang 14068, Korea; 5 Department of Otorhinolaryngology-Head & Neck Surgery, Sacred Heart Hospital, Hallym University College of Medicine, Anyang 14068, Korea; 6 Department of Oral and Maxillofacial Surgery, Dongtan Sacred Heart Hospital, Hallym University College of Medicine, Dongtan 18450, Korea; 7 Department of Otolaryngology-Head & Neck Surgery, Dongtan Sacred Heart Hospital, Hallym University College of Medicine, Dongtan 18450, Korea; 8 Department of Oral & Maxillofacial Surgery, Korea University Guro Hospital, Seoul 08308, Korea; ungassi@naver.com

**Keywords:** 3D printing, hydroxyapatites, beta-tricalcium phosphate, HA/TCP, recombinant human bone morphogenetic protein-2

## Abstract

This study aimed to evaluate the bone regeneration capacity of a customized alloplastic material and xenograft with recombinant human bone morphogenetic protein-2 (rhBMP-2). We prepared hydroxyapatite (HA)/tricalcium phosphate (TCP) pure ceramic bone blocks made using a 3D printing system and added rhBMP-2 to both materials. In eight beagle dogs, a total of 32 defects were created on the lower jaws. The defective sites of the negative control group were left untreated (N group; 8 defects), and those in the positive control group were filled with particle-type Bio-Oss (P group; 12 defects). The defect sites in the experimental group were filled with 3D-printed synthetic bone blocks (3D group; 12 defects). Radiographic and histological evaluations were performed after healing periods of 6 and 12 weeks and showed no significant difference in new bone formation and total bone between the P and 3D groups. The 3D-printed custom HA/TCP graft with rhBMP-2 showed bone regeneration effects similar to that of particulate Bio-Oss with rhBMP-2. Through further study and development, the application of 3D-printed customized alloplastic grafts will be extended to various fields of bone regeneration.

## 1. Introduction

Insufficient alveolar bone volume is usually caused by significant bone resorption after tooth extraction. Bone loss after extraction occurs mainly on the ridge’s buccal surface within the first three months. After two years, the horizontal and vertical widths of the alveolar ridge decrease by 40–60% on average [1,2,3,4]. In addition to the extraction, the alveolar ridges may be lost due to surgical resection or congenital defects. Since the appropriate volume of the alveolar bone is crucial for successfully restoring prosthesis and implant, the reconstruction of the resorbed alveolar ridge is an important goal for clinicians. Therefore, bone grafting was developed to facilitate further treatment for patients in need of implants and to provide favorable results [5,6].

An autogenous bone graft is considered the best option because of its high bone-inducing ability and low infection risk [7,8]. The autogenous bone graft is harvested from an adjacent or distant donor site within the same patient to reconstruct the deficiencies. The limited amount of bone harvest, postoperative pain, difficulty in daily activities, and morbidity at the donor site triggered a search for non-autogenous tissue [9]. Interest in research on bone substitutes is increasing, and various products are being manufactured and used for clinical treatment.

An allograft is taken from the same species’ cadaver and then treated to eliminate the possibility of infection and antigen reaction. Allografts are provided in powder or block forms from a specially designated organizing bank. A xenograft comprises bone tissue derived from non-human subjects. To eliminate antigenicity caused by differences between species, xenografts are made of pure calcium ceramic from which all organic components have been removed. Deproteinized bovine bone minerals are widely used, such as Bio-Oss^®^ (Geistlich Pharma AG, Wolhusen, Switzerland). Graft materials from a living body may have the potential for disease transmission or immunologic rejection. Therefore, these materials are exposed to X-rays, freezing, and chemical processes to prevent this, thereby reducing osteogenesis capacity.

Alloplastic graft material is a pure industrially synthesized bone substitute that is not obtained from a living body. Various materials in which calcium and other elements are combined are used, such as bioactive glass, hydroxyapatite (HA), tricalcium phosphate (TCP), and calcium sulfate [10].

However, bone substitutes cannot be entirely superior to autogenous bone because of the bone morphogenetic proteins (BMPs), which are bone-inducing substances in the natural bone matrix. The addition of growth factors, such as recombinant human bone morphogenetic protein-2 (rhBMP-2) and polydeoxyribonucleotide (PDRN), can improve the biological activity and results [11,12,13,14]. A derivative of bone and cartilage formation, rhBMP-2 was first identified in the 1960s and was approved in 2007 as an alternative to an autogenous bone graft for the maxillary sinus, closure surgery of a cleft palate, and localized alveolar ridge augmentation [12,15,16,17]. In several studies, the application of rhBMP-2 accelerated new bone formation in the bone defects [18,19,20,21]. PDRN is a material used to improve tissue regeneration capacity. It contains nucleosides extracted from deoxyribonucleic acid obtained from the sperm of salmon trout, from which the active protein or peptide has been removed through purification and sterilization [22]. It has also been demonstrated to stimulate the repair of tissue lesions and act as a growth stimulator for fibroblasts, osteoblasts, endothelial cells, and glial cells [23,24].

Recently, advances in computer-aided design and manufacturing (CAD/CAM) technology have made it possible to use customized compositions or shapes of alloplastic bone graft materials [25,26,27]. Furthermore, it is expected that a technology that analyzes bone defects in three-dimensions (3D) and manufactures materials into various shapes will be gradually introduced [28,29,30]. Three-dimensional printing, a machining process for creating a 3D scaffold, can help produce several products at once with less material. The convenience of manufacturing can significantly improve clinical efficiency. Electrospun polymer nanofibers, such as poly L-lactic acid and polycaprolactone, have been 3D-printed and used as alloplastic bones; however, these have poor biocompatibility, a low bone formation rate, and poor absorption. Therefore, ceramic bone grafts customized from 3D printing have recently been developed [31,32].

Preference between alloplastic and bovine-derived natural substances may vary from patient to patient. Since standardized histological comparisons are rare, choosing between the two materials can be difficult for patients and clinicians. Furthermore, there have been few studies that compared alloplastic materials with growth factors and bovine-derived materials with growth factors, such as BMP or PDRN [12]. This study aimed to compare the bone regeneration capacity of xenograft materials and customized allograft materials 3D printed in a block shape with rhBMP-2.

## 2. Results

### 2.1. Architecture of Scaffold

**Figure 1:**
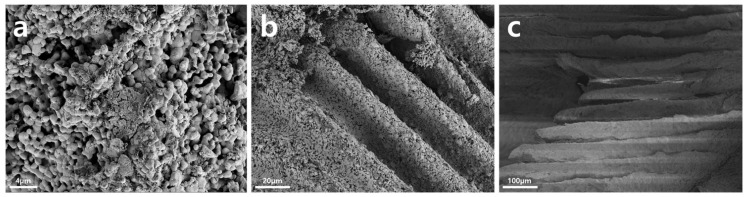
The architecture of 3D-printed HA/TCP scaffolds (SEM): (**a**) ×2000; (**b**) ×500; (**c**) ×100.

The scaffolds were printed with a resolution of 100 μm and a thickness of 20–100 μm. The architecture of the 3D-printed scaffold was regular and sturdy. The architecture of the 3D-printed HA/TCP scaffold was analyzed using microscopy tests (Figure 1).

### 2.2. Cytotoxicity Test

After 24 and 48 h of culturing, the rating of the cells using negative control elution was zero, and that of the cells using positive control elution was four, showing suitable conditions (Figure S1). The elution of the control remained unchanged. The cell rating of the HA/TCP sample was zero for 24 and 48 h, indicating that it was not cytotoxic.

### 2.3. Clinical Findings

During the observation period, no infection or other abnormal symptoms were clinically observed at the surgical site, and the beagles clinically showed no specific signs of morbidity. Moreover, 6 and 12 weeks later, no lesions or abnormalities were observed in the alveolar and mandibular bones at the time of the euthanasia.

### 2.4. Radiological Evaluation

**Figure 2:**
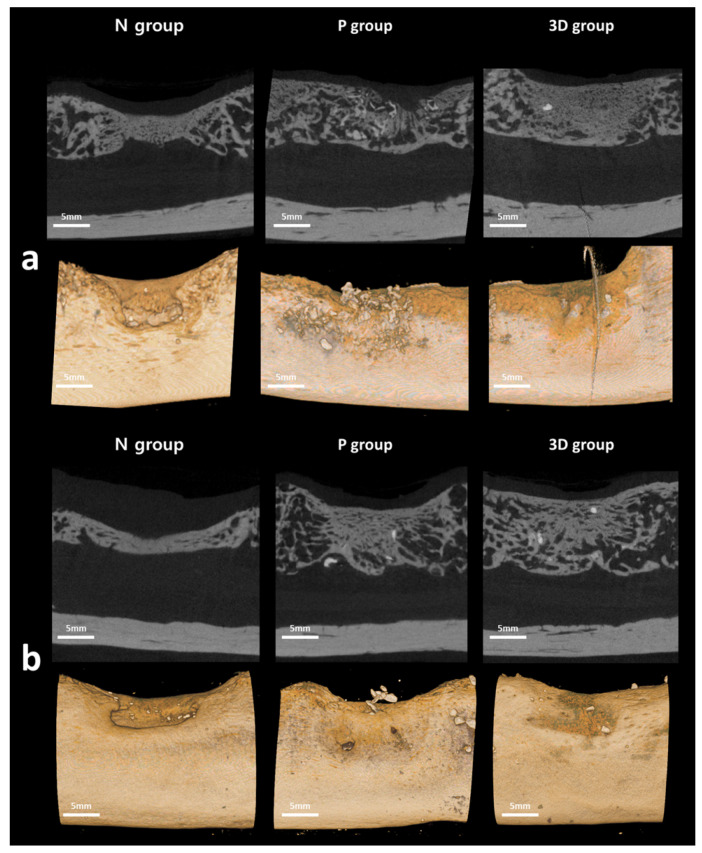
Radiological evaluation at the surgical site of each group at 6 and 12 weeks. At 6 and 12 weeks, the N group showed less bone regeneration at the defect site than the P and 3D groups (N group = negative group without graft, P group = positive group grafted with Bio-Oss, 3D group = study group grafted with 3D-printed ceramic block): (**a**) 6 weeks; (**b**) 12 weeks.

The radiological examination was performed to calculate the new bone formation rate after 6 and 12 weeks (Figure 2) (Table 1).

The radiographic analysis after six weeks showed that the rate of new bone formation was highest in the P group (32.63), followed by the 3D (29.57) and N (21.57) groups. Except for the difference between the P and N groups (*p* = 0.010), the difference in size between each group was not statistically significant (*p* > 0.05). Furthermore, the total amount of bone was highest in the P group (34.89), followed by the 3D (30.50) and N (21.57) groups. Additionally, there was a significant difference only between the P and N groups (*p* = 0.033). In the radiographic analysis after 12 weeks, the rate of new bone formation was highest in the P group (45.49), followed by the 3D (43.79) and N (27.51) groups. The difference in size between each group did not show statistically significant differences (*p* > 0.05). Furthermore, the total amount of bone was highest in the P group (49.22), followed by the 3D (45.70) and N (27.51) groups. The difference in size between the P and N groups showed statistically significant differences (*p* = 0.015); when compared with the 3D group, both the N and P groups did not show statistically significant differences (*p* > 0.05).

### 2.5. Histological Evaluation

**Figure 3:**
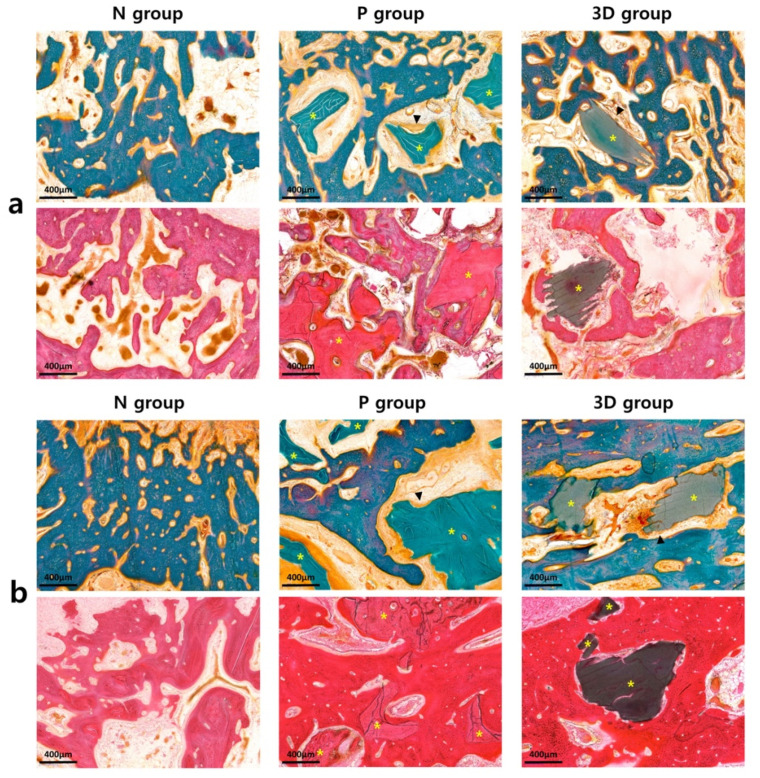
Histological evaluation using hematoxylin and eosin (H & E) staining and Goldner’s trichrome (GT) staining (N group = negative group without graft, P group = positive group grafted with Bio-Oss, 3D group = study group grafted with 3D-printed ceramic block; yellow asterisk: grafted bone substitutes; black arrowhead: osteoblast): (**a**) 6 weeks; (**b**) 12 weeks.

The histological examination was performed to calculate the new bone formation rate after 6 and 12 weeks (Figure 3) (Table 2).

When comparing the differences between the groups after six weeks, the proportion of the new bones was highest in the P group (25.25), followed by the 3D (23.90) and N (21.56) groups. However, the difference between the three groups was not statistically significant (*p* > 0.05). The total bone ratio, combined with the new bone and graft ratio, was the highest in the P group (32.38), followed by the 3D (27.81) and N (21.56) groups. The difference between the 3D group and the N and P groups was not statistically significant (*p* > 0.05). However, there was a statistically significant difference between the N and P groups (*p* = 0.002). When comparing the differences between groups after 12 weeks, the proportion of new bones was highest in the 3D group (39.54), followed by the P (30.54) and N groups (25.42). However, the difference between the three groups was not statistically significant (*p* > 0.05). The total bone ratio was the highest in the 3D group (43.01), followed by the P (39.32) and N (25.42) groups. The difference between the 3D and P groups was not statistically significant (*p* > 0.05). However, there was a statistically significant difference between the N and 3D groups (*p* = 0.041) and between the N and P groups (*p* = 0.015).

## 3. Discussion

Autogenous bone is the gold standard for bone graft material, but there are problems with the limit of the amount and complications related to donor site defects [33]. To solve this issue, continuing efforts are being made to find an optimal bone substitute that has bone generation capability, maintains the alveolar bone shape for a certain period, and then degrades properly in a biological environment. This study compared and evaluated the degree of bone regeneration by using Bio-Oss and alloplastic bone customized by 3D printing in relatively large defects. The same amount of rhBMP-2 was added to both materials to improve the bone induction ability of the alloplastic bone. As of 2021, a few papers indexed on PubMed have demonstrated which type of bone graft further enhances the effect of the rhBMP-2 by comparing the difference in bone formation ability. This is the first report comparing customized alloplastic bone and xenografts’ bone regeneration ability by applying an rhBMP-2 in the same amount. Moreover, the results of this study confirm that there was no significant difference between the two graft materials when applying the rhBMP-2.

Although various studies and clinical results have demonstrated the effectiveness of rhBMP-2, concerns about the safety of its widespread use have also been raised. First, there are several studies on the correlation between the use of rhBMP-2 and edema [34,35]. Additionally, there are controversies in the orthopedic literature about the possible increase in the risk of malignancies due to the use of rhBMP-2, some of which include an increased risk [36,37] and others with no association [38]. Tannoury et al. reviewed the orthopedic literature and summarized a wide range of side effects associated with rhBMP-2 use in the lumbar and cervical spine as follows. Neurological diseases may occur, such as postoperative radiculitis or nerve root injury, ectopic bone formation or osteolysis, postoperative edema-related dysphagia, neck swelling, and hematoma formation [39]. It is also necessary to consider the dosage capacity, cost-related issues, carrier types, and theoretical carcinogenesis concerns [39,40].

Bone regeneration using Bio-Oss and bovine bone has been recognized for its stability as a treatment for alveolar and craniofacial bone defects over the past few years [41,42,43]. In a study in dogs, when Bio-Oss was grafted in the extraction socket, the ridge improved after six months compared to the ungrafted sites [44]. This natural bone substitute promotes bone growth in human bone defects and breaks down very slowly through metabolism after grafting [45,46]. In general, Bio-Oss is known to be superior when comparing the bone formation ability of alloplastic bone and Bio-Oss [47]. However, in Bio-Oss, the medical cost of materials is very high, so there may be economic problems.

Furthermore, it is difficult to apply this method to a pervasive defect. Additionally, 3D printing cannot be applied in xenogenic grafts, such as Bio-Oss; it must be cut using a milling machine for customized production. This is less accurate than 3D printing, and there is unnecessary loss during milling and a problem in which the margin is easily broken [48,49]. For these reasons, there are no customized xenograft products for clinical use. Therefore, alloplastic bones that can be applied to a wide range of defects and customized for patients by 3D printing are being actively researched and developed. Their use in dental clinics is increasing.

The alloplastic bone used in our study is a ceramic bone substitute consisting of a combination of HA and β-tricalcium phosphate (β-TCP). Hydroxyapatite, the main mineral component of bone, is used for various purposes, such as alveolar bone grafting and ridge preservation. It shows resistance to physiological absorption due to the low solubility of calcium phosphate at physiological pH [50]. The β-TCP is proposed as an osteoconductive material that can provide a matrix for new bone deposition. Unlike HA, the β-TCP is resorbed and replaced by new bones. However, as its absorption is not supplemented by bone generation at the same rate, bone formation is less than the volume of β-TCP. Therefore, combining it with insoluble HA allows β-TCP to promote bone regeneration while HA maintains space.

The mechanical strength of 3D-printed alloplastic bone is lower than that of natural human bone. Many studies are being conducted to increase the mechanical strength and bone regeneration ability of 3D-printed bones to overcome this difference. In Lim et al., the compressive strength of 3D-printed bones of various designs was compared, and the cubic design showed greater compressive strength than the diamond design with the same pore size [32]. High compressive strength can help the prognosis in bone regeneration. However, the mechanical strength and decomposition of the scaffold were compared with the structure and porosity according to Kolan et al. In this study, the diamond-structured scaffold showed better results. There was no significant difference in mineralized bone formation between the cubic and diamond scaffold implanted defects, but a higher percentage of fibrous connective tissue and osteoblast activity was found at the diamond treatment site [51].

In this study, particle-type xenogenic bone was used as a positive control group (P group) compared with the 3D-printed block-type alloplastic bone study group (3D group). Before the experiment, we hypothesized that there would be more residual graft material in the defects treated with the block-type graft material. This is because it firmly supports the surrounding tissue and maintains its original volume and shape, almost without the loss of bone substitute particles. Most studies comparing block- and particle-type bone substitutes have shown that the block-type absorption rate is lower. The study by Dasmah et al. confirmed that the change in the particulate bone graft site tended to be greater than that of the block bone after two years of the autologous bone graft [52].

Similarly, in a study by Benic et al., it was found that the block bone substitute used for the guided bone regeneration of peri-implant defects showed a better hard tissue increase rate than the particulate bone substitute after six months of healing [53]. However, in our study, there was no significant difference between the total bone volume in the 3D and P groups after 6 and 12 weeks. The causes can be estimated as follows.

First, since the size of the defect was not large, the difference between the two types of grafts may not have been significant. The more extensive defects would have resulted in more loss of graft particles. Second, the difference between the two grafts was offset by the addition of the rhBMP-2, a potent derivative of bone. Another cause may be that the particle-type material is filled more densely than the porous block customized to the size of the defect. Finally, in this study, the screw was not fixed to the ceramic block because of fracture of the graft material due to torque during screw fixation. In addition, no membrane was applied after the grafting. Therefore, there may have been mobility and loss of the block graft material.

This study had several limitations. First, the block graft of the 3D group was not fixed as mentioned above. Therefore, it was not possible to confirm the bone regeneration effect of the block-type graft material in a perfectly fixed condition. Second, in the repair procedure of both particle- and block-type materials, the mastication or tongue movement could not be controlled and may have been affected. Third, since we were targeting beagles of the same age and size, the study was based on the premise that the conditions for vascularization were the same. However, the vascularization could differ for individuals and for each defect within an individual, thereby affecting the results. Fourth, a complete necropsy was not performed after euthanasia. Therefore, the side effects of rhBMP-2, such as toxicity to other organs, could not be accurately identified. However, since only a small dose of rhBMP-2 was locally applied to the graft materials, it was assumed that the possibility was very low. Moreover, there were no clinical problems observed in the animals. Finally, histological evaluation of all areas of the bone is difficult. Therefore, evaluating three-dimensional bone formation was difficult only based on our histological findings. Further studies are needed to broaden the application range and increase the effectiveness of 3D-printed HA/TCP scaffolds without polymers.

## 4. Materials and Methods

### 4.1. Subject

The study was conducted on eight beagles. They were 12 months old and weighed about 13.5 kg. Eight weeks before the experiment, the premolars and the first molar of the mandible on both sides were extracted. The sites were healed with a flat edentulous ridge. The animal preparation and surgical protocols were approved by the Animal Ethics Committee for Animal Research (CRONEX-IACUC: 202004003) and according to the ARRIVE and PREPARE guidelines.

### 4.2. Preparation of Scaffolds

**Figure 4:**
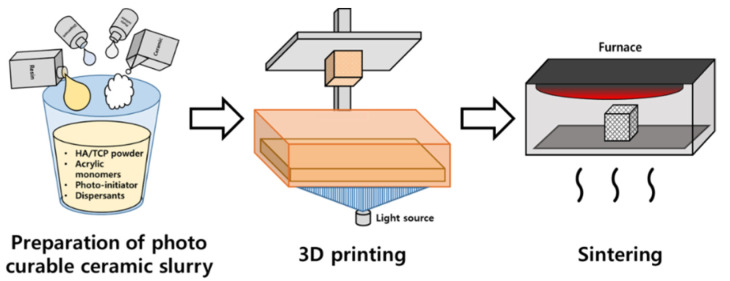
The overall procedure of 3D-printed pure ceramic scaffold fabrication.

A block-shape (9 × 9 × 10 mm^3^) scaffold with diamond pore architecture and pore size of 1.2 mm was designed in a computer program and stored as a stereolithography file for manufacturing purposes. The file was printed on a digital light processing (DLP) 3D printer (Cubicon Lux, Cubicon^®^, Seongnam, Korea) using photocatalytic ceramic resin composite materials. The resolution of the 3D printer is 100 μm and it can print up to a thickness of 20–100 μm. The mixture consisted of a 6:4 ratio HA/TCP (Dentium^®^, Suwon, Korea) and dispersants, acrylic monomers, and photo-initiator (phenyl bis phosphine oxide; Sigma-Aldrich^®^, St. Louis, MO, USA). A photo-reactive ceramic resin composite material was prepared by mixing proprietary resin with 64 wt.% ceramic powder. The mixture was put into the tank of a 3D printer (Cubicon Lux, Cubicon®, Sungnam, Korea) with a transparent bottom, and the blocks were printed via polymerization by projecting ultraviolet rays. Printouts are formed on build plates that move up and down. After printing, the blocks were carefully separated from the build plate, and washed clean with distilled water to remove the residual mixture. We then sintered the blocks for ten hours in a furnace at 1250 °C (Carbolite, Ubstadt-Weier, Germany) to remove the resin polymer. The entire process is shown in a schematic diagram (Figure 4).

A pure ceramic scaffold without resin polymer was obtained. Scanning injection electron microscopy (SEM) images were acquired on a Zeiss Sigma HD (Zeiss, Jena, Germany) with an accelerating voltage of 2–8 kV at different magnifications.

### 4.3. Cytotoxic Tests

The cytotoxicity of the specimen was confirmed using an in vitro cytotoxicity test standard (ISO 10993-5). The sample was eluted from the elution solvent for 24 h at 37 °C. The elution solvent was constructed with minimal essential medium (MEM; 500 mL), 50 mL of fetal bovine serum (Gibco^®^, Thermo Fisher Scientific, Green Island, NY, USA), and 10 mL of penicillin-streptomycin solution (Welgene^®^, Gyeongsan-si, Korea). The extraction was performed at a constant rotation speed of 50 rounds/min. A 4 g specimen was estimated with 20 mL extraction medium of pH 7.4 (0.2 g/mL). The pH was measured and was the same as the extraction medium before extraction. Under the same conditions, elution tests were performed on the experimental solution, with natural rubber as a positive control, and high-density polyethylene as a negative control. Mouse fibroblasts (ATCC CCL 1, clone 929 of strain L, Korean Research Institute of Bioscience and Biotechnology, Daejeon, Korea) were put into a solution flask and cultured in a 37 °C incubator. After one day in the six-well plates containing the cell culture medium, it was verified that cell confluence was more than 80%. After completely draining the cell culture medium, two milliliters of the test and control solutions were poured into each well. The cell growth and lysis levels were examined using microscopes after 24 and 48 h of incubation. If the cells that used negative control elution were rated zero, and those that used positive control elution were grade three or four, it was considered to be a suitable test condition. Achieving a numerical grade greater than 2 was considered indicative of cytotoxicity. The experiment was performed in triplicate.

### 4.4. Surgical Procedures

**Figure 5:**
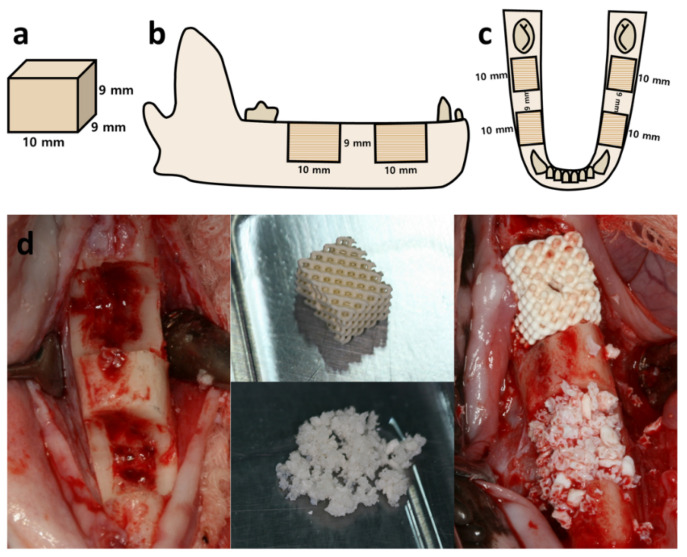
Design of the defect and actual surgical procedure in the mandible of the beagle: (**a**) the shape and size of the defect; (**b**) defect design on the lateral view; (**c**) defect design on the occlusal view; (**d**) surgical procedure.

When the extraction sites healed after eight weeks, an operation was performed under general anesthesia induced by intravenous injection. After intubation, isoflurane (Piramal Critical Care, Mumbai, India.) and oxygen inhalation were continued to maintain anesthesia. The incision was made parallel to the edentulous alveolar ridge and vertically on both sides of the defect. A full-thickness buccal flap with mesial relief incision and lingual flap was separated from the alveolar bone. The guide designed to fit the critical defect size (9 × 9 × 10 mm^3^) was then adapted, and the defect was marked with a pencil on the cortical bone, which was made by a preparation using a low-speed denture bur. Two defects were formed on each side of the mandible of the beagle, resulting in a total of 32 defects (Figure 5b,c). The defects were divided into three groups and grafted according to each group. The sample size was determined based on a similar previous study [54]. The study group (3D group, 12 defects) was assigned the grafted allogenic bone blocks, 3D-printed to the size of the defect, and the positive control group (P group, 12 defects) was assigned with the same volume of the grafted xenogenic bone (Bio-Oss) in the form of particles (Figure 5).

All bone materials were hydrated with 0.3 mL of rhBMP-2 at a 0.2 g/mL concentration for each specimen. No graft material was applied to the negative control group (N group, eight defects) for spontaneous bone healing. The flap was adapted and sutured using absorbable thread, 4–0 Vicryl^®^ (Ethicon, Somerville, NJ, USA). The removal of sutures was performed two weeks after surgery. Animals were observed weekly until euthanasia, and conditions of the surgical site such as infection, inflammation, and dehiscence were confirmed. For analysis of the mandible, four of the beagles were euthanized six weeks after the surgery, while the other four were euthanized 12 weeks later. Bone formation in this experiment would be similar to that in bone fracture healing. For fractures, a hard callus forms at about 6 weeks, and bone remodeling occurs after 8 weeks. Considering this theory, we decided that the observation period should be between 6 and 12 weeks. We did not perform a complete necropsy to confirm if there was damage to other organs.

### 4.5. Analysis

#### 4.5.1. Radiological Examination

The extracted mandibular bone was subjected to micro-computed tomography (SkyScan1173^®^, Bucker-CT, Kontich, Belgium). The following conditions were taken: 130 kVp tube voltage, 60 μA tube current, 1 mm aluminum filter, 500 ms exposure time, and 0.3° rotation angle. A total of 800 images were acquired with a pixel size of 13.85 mm, and the number of pixels was 2240 × 2240. NRecon (Bruker-CT, Kontich, Belgium) was used for cross-sectional reconstruction, and Data Viewer (Bruker-CT, Kontich, Belgium) and Ct-VOX (Bruker-CT, Kontich, Belgium) were used for the 3D reconstruction. The bone volume was identified by the intensity value ranging from 55 to 255, and the following formula was used to calculate the newly formed bone:

(1) Percent bone volume (%)=[Bone volume/Tissue volume]×100.

#### 4.5.2. Histological Examination

A single investigator who was blinded to the groups performed all the histological analyses. The extracted mandible was preserved in formalin for a week. The formalin-fixed mandible was then washed with running water for nine hours and cut to prepare tissue slides. Hematoxylin and eosin or Goldner’s trichrome were used for tissue staining. A total of 64 slides were prepared, two for each type of staining technique. The images of the tissue slides were recorded in an objective lens with a magnification of ×1.25 and ×4 using optical microscopes (OLYMPUS BX50^®^, Olympus Optical CO. Tokyo, Japan), and at ×10 and ×20 magnification for high resolutions. The percentages of the newly formed bone and total amount of bone were analyzed using Image-Pro Plus^®^ (Media Cybernetics, Rockville, MD, USA), and calculated using the following formulae:

(2) Percentage of new bone (%)=[Area of new bone/Total area of defect]×100

(3) Percentage of remaining graft (%)=[Area of graft/Total area of defect]×100

(4) Percentage of total bone (%)= Percent new bone + Percent remaining graft

#### 4.5.3. Statistical Analysis

In this study, the observations in each group came from populations with distributions of the same shape, and the samples were random and independent. Therefore, Kruskal–Wallis testing was performed to analyze the differences between the three groups; Mann–Whitney U tests were used for comparison between the groups. A *p*-value < 0.05 was considered statistically significant, and statistical analysis was performed using the SPSS program (Version 12.0K, SPPS, Inc., Chicago, IL, USA).

## 5. Conclusions

The 3D printing of HA/TCP scaffolds via the DLP system is an unprecedented approach that would allow customized bone grafting for in vivo applications. New bone formation in the case of 3D-printed customized HA/TCP scaffolds was not significantly different from particulate Bio-Oss when combined with rhBMP-2.

## Figures and Tables

**Table 1:** Radiological analysis of new bone formation in each group at 6 and 12 weeks.

		N Group	P Group	3D Group	Difference (*p*)
6 weeks	New bone (%)	21.57 ± 1.86	32.63 ± 7.06	29.57 ± 9.41	0.041 *
	Total bone (%)	21.57 ± 1.86	34.89 ± 6.25	30.50 ± 9.81	0.033 *
12 weeks	New bone (%)	27.51 ± 14.22	45.49 ± 12.09	43.79 ± 19.35	0.073
	Total bone (%)	27.51 ± 14.22	49.22 ± 14.33	45.70 ± 19.39	0.039 *

The *p* value from the Kruskal–Wallis test; average ± standard deviation; * statistical significance at *p* < 0.05.

**Table 2:** Histological analysis of new bone formation in each group at 6 and 12 weeks.

		N Group	P Group	3D Group	Difference (*p*)
6 weeks	New bone (%)	21.56 ± 1.84	25.25 ± 6.49	23.90 ± 9.57	0.365
	Total bone (%)	21.56 ± 1.84	32.38 ± 7.68	27.81 ± 6.73	0.013 *
12 weeks	New bone (%)	25.42 ± 14.27	30.54 ± 14.92	39.54 ± 7.83	0.065
	Total bone (%)	25.42 ± 14.27	39.32 ± 12.16	43.01 ± 8.23	0.026 *

The *p* value from the Kruskal–Wallis test; average ± standard deviation; * statistical significance at *p* < 0.05.

## Data Availability

No new data were created or analyzed in this study. Data sharing is not applicable to this article.

## Supplementary Materials

The following are available online at https://www.mdpi.com/article/10.3390/ijms222212518/s1.
