# Supplementary figures and images for: Bone Regeneration of a 3D-Printed Alloplastic and Particulate Xenogenic Graft with rhBMP-2
Source: Int J Mol Sci. 2021 Nov 20;22(22):12518. doi: 10.3390/ijms222212518 (PMC8624569; doi:10.3390/ijms222212518)

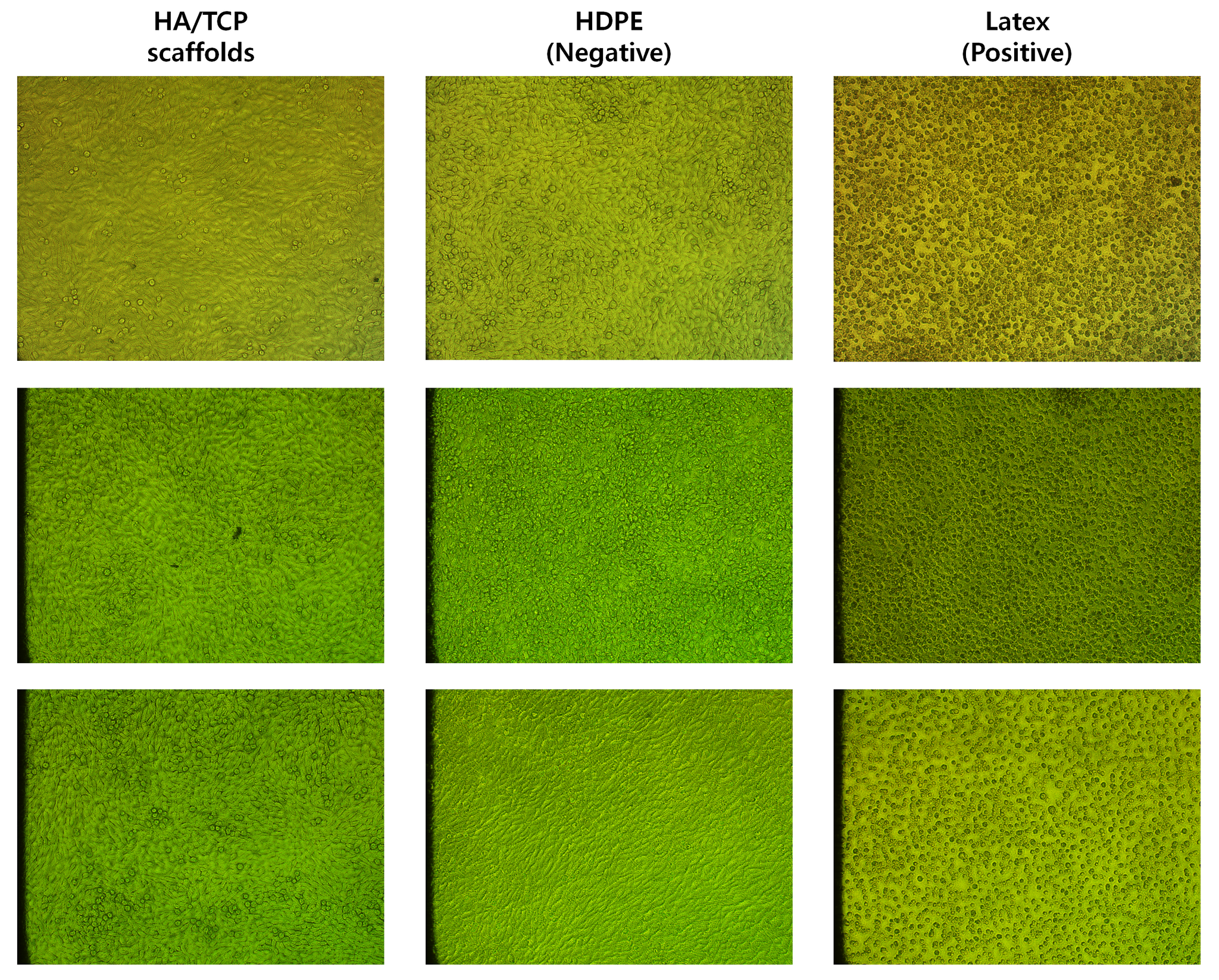

Supplement: Supplementary file 1 [file ijms-22-12518-s001.zip › ijms-1441600-supplementary.tif]
